# Supplementary material for: Comparison of different methods for monitoring changes in bone mineral density during follow-up measurements
Source: PLoS One. 2026 Feb 11;21(2):e0339343. doi: 10.1371/journal.pone.0339343 (PMC12893572; doi:10.1371/journal.pone.0339343)
Supplement: Attachment — Submitted filename: Response to the reviewer.docx [file pone.0339343.s002.docx]

**Comments to the Author**

1. Is the manuscript technically sound, and do the data support the conclusions?

The manuscript must describe a technically sound piece of scientific research with data that supports the conclusions. Experiments must have been conducted rigorously, with appropriate controls, replication, and sample sizes. The conclusions must be drawn appropriately based on the data presented.

Reviewer #1: Partly

**Response:** Thank you for your comment. Our aim is to emphasize the need for structured interpretive frameworks that help clinicians understand and respond to discordant findings, rather than to suggest equivalence or direct comparisons between measurement sites or between BMD and TBS. To clarify this distinction, we have revised the manuscript to avoid implying that such discordance is inherently negative. Instead, we highlight that these findings underscore the importance of site- and modality-specific interpretation in the longitudinal monitoring of bone health. This issue is especially relevant in routine follow-ups, where treatment decisions often hinge on the perceived response to therapy. We believe that recognizing this interpretive ambiguity further supports the need for clearer clinical guidance. In response, we have clarified our discussion, incorporated additional relevant literature, and conducted a further round of English language editing to improve clarity and readability. We hope these revisions enhance the scientific contribution of our work.

2. Has the statistical analysis been performed appropriately and rigorously?

Reviewer #1: Yes

**Response:** Thank you for your comment.

3. Have the authors made all data underlying the findings in their manuscript fully available?

The [PLOS Data policy](http://track.editorialmanager.com/CL0/http:%2F%2Fwww.plosone.org%2Fstatic%2Fpolicies.action%23sharing/1/010f01975d869194-40ef4d20-eb92-4df1-bcf3-ead4490f73c2-000000/4IvNGNlABDIlAsOVG-_DaQPsLryESQibFJWEWD0A9Bg=215) requires authors to make all data underlying the findings described in their manuscript fully available without restriction, with rare exception (please refer to the Data Availability Statement in the manuscript PDF file). The data should be provided as part of the manuscript or its supporting information, or deposited to a public repository. For example, in addition to summary statistics, the data points behind means, medians and variance measures should be available. If there are restrictions on publicly sharing data—e.g. participant privacy or use of data from a third party—those must be specified.

Reviewer #1: No

**Response:** Thank you for your comment. The data set is owned by the institutional review board of Cheng Hsin General Hospital. The institutional review board of Cheng Hsin General Hospital only approved the data analysis in our study and did not approve data sharing. Therefore, we do not have permission to share the data set. Interested researchers can submit data access requests to the institutional review board of Cheng Hsin General Hospital through the following email address: chghirb@chgh.org.tw Others would be able to access the data in the same manner as the authors. We have included the data availability statement on page 18, line 316.

**Page 18, line 316**

**Data Availability statement:**

The dataset used in this study is owned by the Institutional Review Board (IRB) of Cheng Hsin General Hospital. While the IRB approved our use of the data for analysis, it did not grant permission for data sharing. As a result, we are unable to make the dataset publicly available. However, interested researchers may request access by contacting the IRB of Cheng Hsin General Hospital at chghirb@chgh.org.tw. Access will be provided under the same conditions as those granted to the authors, who do not have any special privileges.

4. Is the manuscript presented in an intelligible fashion and written in standard English?

PLOS ONE does not copyedit accepted manuscripts, so the language in submitted articles must be clear, correct, and unambiguous. Any typographical or grammatical errors should be corrected at revision, so please note any specific errors here.

Reviewer #1: Yes

**Response:** Thank you for your comment.

5. Review Comments to the Author

Please use the space provided to explain your answers to the questions above. You may also include additional comments for the author, including concerns about dual publication, research ethics, or publication ethics. (Please upload your review as an attachment if it exceeds 20,000 characters)

Reviewer #1: This manuscript reports on methods to assess and evaluate changes in DXA derived bone outcomes over time. In particular, differences in hip BMD when the mean of both hips is analyzed vs both hips separately, differences in change between hip and LS BMD, and differences in change between LS BMD and TBS. Generally, the study seems well conducted, although I do not fully agree on the interpretation of the data.

1. Generally:
Sometimes the story is difficult to follow, especially the hip comparison. The authors use: mean bilateral total hip BMD vs. total hip BMD of both hips. I would recommend using: mean bilateral total hip BMD vs. total hip BMD of each hip separately.

**Response:** Thank you for your comment. We have revised the manuscript on page 2, line 20, page 4, line 66, and page 5, line 86.

**Page 2, line 20**

Monitoring bone mineral density (BMD) changes can be challenging when both the spine and hips are included in follow-up assessments. This study aimed to evaluate BMD changes using different monitoring approaches, including comparisons between the mean bilateral total hip BMD and total hip BMD of each hip separately, spinal BMD and mean bilateral total hip BMD, and spinal BMD and trabecular bone score (TBS).

**Page 4, line 66**

The International Society for Clinical Densitometry (ISCD) recommends that when both hips are scanned on repeat tests, the mean bilateral total hip BMD should be used for monitoring [2]. However, the differences between the monitored mean bilateral total hip BMD and the total hip BMD of each hip separately are uncertain.

**Page 5, line 86**

Therefore, the aim of this study was to compare different monitoring methods (mean bilateral total hip BMD vs. total hip BMD of each hip separately, spinal BMD vs. mean bilateral total hip BMD, spinal BMD vs. TBS); additionally, we also aimed to determine LSC when monitoring mean bilateral total hip BMD in follow-up BMD measurements.

2. Introduction:
“However, the ISCD does not mention how to interpret BMD changes when both the regions are scanned during follow-up BMD measurements. Therefore, discordant results may be obtained when simultaneously monitoring BMD changes in the spine and hips.”
Is this really a problem? There are many factors involved in BMD changes over time. For example, different (un)loading patterns of the spine and hip may strongly influence the magnitude and direction of change for both sites.
General for entire introduction: Yes there can be discordance in the magnitude or direction of change between BMD of different scans or different sites. But I am not sure whether this is really important. In many case, the discordance can be explained by specific loading patterns or interventions that can affect some sites or outcomes more than the other. This also justifies the rationale to conduct multiple scans. The interpretation of the results can than be based on multiple scans….
The theoretical framework would be strengthened by a more comprehensive integration of the relevant literature.

**Response:** We appreciate the reviewer’s thoughtful observation regarding the discordance in BMD changes between different skeletal sites, such as the spine and both hips. Indeed, as correctly noted, there are several factors—including mechanical loading patterns, pharmacological interventions, and degenerative changes—that can contribute to differential changes in BMD at these sites over time. This variability is one of the reasons why multiple-site assessment is recommended in clinical practice.

However, our point was not to question the need for multiple-site measurements, but rather to highlight a gap in current clinical guidelines. Specifically, the ISCD does not provide explicit guidance on how to interpret discordant longitudinal BMD changes when the spine and both hips are assessed during follow-up. While individual site changes can often be explained retrospectively, there is limited standardization or consensus on how to weigh such differences when making clinical decisions—particularly in cases where one site shows improvement while another shows deterioration.

This lack of standardized interpretation can be especially relevant in clinical trials or routine follow-ups where treatment decisions (e.g., initiation or discontinuation of osteoporosis therapy) hinge on the perceived response. As such, we believe that acknowledging this ambiguity reinforces the need for clearer interpretive frameworks in longitudinal BMD monitoring and justifies our focus on this issue. We have revised the manuscript on page 4, line 73.

**Page 4, line 73**

Various factors such as mechanical loading, pharmacological interventions, and degenerative changes can lead to site-specific variations in BMD over time, supporting the use of multiple-site assessments in clinical practice. The ISCD recommends measuring BMD at both the spine and hip for all patients [2]. However, the ISCD does not provide clear recommendations for interpreting discordant BMD changes when the spine and hips are assessed during follow-up. While such differences can sometimes be explained retrospectively, there is no standardized approach for incorporating them into clinical decision-making, especially when one site improves and another worsens. This ambiguity is particularly relevant in settings where treatment decisions rely on BMD trends.

The ISCD also recommends evaluating the bone quality using the trabecular bone score (TBS) [4]. When monitoring changes in the spinal BMD and TBS simultaneously on repeat tests, practitioners are often uncertain about the association between monitoring spinal BMD and TBS. Therefore, the aim of this study was to compare different monitoring methods (mean bilateral total hip BMD vs. total hip BMD of each hip separately, spinal BMD vs. mean bilateral total hip BMD, spinal BMD vs. TBS); additionally, we also aimed to determine LSC when monitoring mean bilateral total hip BMD in follow-up BMD measurements. Our aim was to highlight a gap in current guidelines, and our focus on this issue emphasizes the need for clearer guidance in interpreting longitudinal BMD data.

3. Methods:
Cohort: What was the median or mean follow-up time?

**Response:** Thank you for your comment. We have revised the manuscript on page 6, line 101.

**Page 6, line 101**

Finally, 1,105 participants were enrolled in the study and the mean follow-up time was 23 months.

4. BMD changes have been reduced to an ordinal scale. Have the authors also considered to address the agreement between changes on a continuous scale? In this case, also the magnitude and direction of changes between outcomes can be assessed.

**Response:** We thank the reviewer for this insightful comment. In our study, we primarily categorized BMD changes using an ordinal scale (e.g., decreased, no change, increased) to support clinical interpretation and to align with commonly used thresholds in practice, such as the least significant change (LSC). The direction of change is also effectively captured using this approach. While continuous scales can provide additional detail, averaging BMD values—such as from both hips—can have a statistical dampening effect, potentially attenuating the apparent magnitude of changes and reducing sensitivity compared to site-specific assessments. Additionally, our intention was not to directly compare the absolute magnitude of change between sites, but rather to point out the clinical challenge of interpreting discordant changes when both are routinely monitored in follow-up assessments. For these reasons, we focused our primary analysis on the ordinal scale, given its clinical relevance and the limitations associated with averaging on a continuous scale.

5. Results:
Table 1: Can you also provide T and Z-scores for sites? This provides an indication how participants’ BMD can be interpreted relative to general population. Was it a cohort characterized by osteoporosis?

**Response:** Thank you for your comment. This study included only postmenopausal women, for whom the International Society for Clinical Densitometry (ISCD) recommends using T-scores rather than Z-scores [Ref]. Accordingly, we have reported T-scores for each site and revised Table 1 to reflect this. The observed prevalence of osteoporosis was 70.1%, which is likely attributable to the hospital-based nature of the study population.

[Reference]. The International Society for Clinical Densitometry (2023) ISCD Official Positions—Adult. <https://iscd.org/official-positions-2023/>.

6. Table 2: Table legend needs to be improved. I assume that the symbol between brackets indicate the right and left hip? This is nowhere explicitly mentioned.

**Response:** Thank you for your comment. Accordingly, we have revised Table 2.

7. Table 2: Alternative explanation: When using the mean of both hips, 5.5% shows no change, while a decrease would have been detected if both hips were analyzed separately. When using the mean of both hips, 4.8% shows no change, while an increase would have been detected if both hips were analyzed separately. In other words, 10.3% (+1.0%) of the participants has and undetected change when the mean of both hips is analyzed, compared with both hips separately. Imo, this shows that analyzing both hips separately is more sensitive for changes than using the mean. But this cannot be regarded as poor agreement.

**Response:** Thank you for your comment. We have revised the manuscript on page 9, line 156 and page 14, line 231.

**Page 9, line 156**

In other words, 11.3% of the participants had an undetected change when monitoring the mean bilateral total hip BMD, compared with both hips separately. This indicates that analyzing both hips separately is more sensitive for changes than using the mean bilateral total hip BMD.

**Page 14, line 231**

Our results revealed that the discordance percentage was 11.3% between the monitored mean bilateral total hip BMD and the individual total hip BMDs (right and left hips). Among the discordant results, 4.8% of the participants had significantly decreased BMD at one side of the hip and no significant change in the mean bilateral total hip BMD. Additionally, 1.0% of the participants had significantly increased BMD involving one hip and decreased BMD at the other hip, which was not significant when monitoring the mean bilateral total hip BMD. This suggests that analyzing each hip separately is more sensitive to detecting changes than using the mean bilateral total hip BMD. Relying solely on the mean may result in overlooking patients who require more aggressive management. While monitoring the mean bilateral total hip BMD is a simpler and more convenient approach for tracking changes in both hips, it can potentially mask significant changes occurring in one hip. Although this method is appropriate for most patients, in clinical situations where greater sensitivity to localized changes is needed—such as during early treatment response or in high-risk individuals—it may be more appropriate to monitor each hip separately. These findings underscore the importance of adopting tailored monitoring strategies based on the clinical context.

8. Table 3: Imo, it does not really does not really make sense to compare the hip with LS. Both sites respond differently to similar loading stimuli and interventions. The outcomes should also be interpreted as such. Anyhow, it does not mean poor agreement.

**Response:** Thank you for your comment. In our study, we used the term “agreement” to describe the concordance or discordance in their longitudinal change patterns during follow-up. We are specifically reporting the statistical measure of Cohen’s kappa. We agree that the lumbar spine and hip respond differently to mechanical loading and therapeutic interventions, and that these differences should be acknowledged when interpreting BMD changes. However, our intention was not to directly compare the magnitude of change between the two sites, but rather to point out the clinical challenge of interpreting discordant changes when both are routinely monitored in follow-up assessments.

In practice, both the spine and hip are measured precisely because they may offer complementary information. Yet, current guidelines (e.g., ISCD) do not provide clear strategies for interpreting discordant trends, such as when BMD increases at one site and decreases at another. This lack of interpretive guidance can lead to uncertainty in clinical decision-making, particularly regarding treatment efficacy or progression. Therefore, rather than suggesting equivalence or direct comparison between the two sites, our aim is to emphasize the need for structured interpretation frameworks that help clinicians understand and act on such discordant findings when they occur. We have revised the manuscript on page 11, line 180 and page 15, line 248.

**Page 11, line 180**

The discordance percentage was 43.5% between the monitored spine and the mean bilateral total hip BMD in follow-up BMD measurements. Cohen’s kappa coefficient was 0.326, indicating poor agreement. Our study was not to directly compare the magnitude of change between these two sites, but rather to point out the clinical challenge of interpreting discordant changes when both are routinely monitored in follow-up assessments.

**Page 15, line 248**

Chen et al. reported that a diagnosis based on the use of the lumbar spine and one site of hip BMD failed to identify 13.4% to 15.8% of osteoporotic subjects, which may indicate that simultaneously monitoring the spine and both hips represented the best practice for DXA BMD measurements [6]. There was spine-hip discordance between the monitored spine and mean bilateral total hip BMD in follow-up BMD measurements in our study and the discordance percentage was 43.5%. Kolta et al. also reported a significant decrease in the hip BMD, whereas the spinal BMD did not change during a 6-year follow-up [7]. The spine was more affected by age-related degenerative changes than the hip. Liu et al. reported that lumbar osteophytes explained 16.6% of measured changes in the spinal BMD in women [8]. In a study of osteoporotic fractures, women with degenerative lumbar disease showed a 9% to 13% increase in the spinal BMD [9]. Ichchou et al. demonstrated that degenerative lumbar disease could increase spinal BMD by up to 15% in women with severe lumbar osteoarthritis [10]. The causes of the spine-hip discordance may be as follows. First, the peak bone mass and rate of bone loss are not the same for different skeletons [11]. Second, the initial rate of bone loss was greater in the trabecular bone than in the cortical bone in postmenopausal women [12]. Third, spine was more affected by age-related degenerative changes than the hip [8]. Therefore, a significant increase in spinal BMD on repeat testing may result from either true bone gain or degenerative changes. If total hip BMD shows a significant decrease during follow-up, aggressive management may still be warranted despite an apparent increase in spinal BMD. Conversely, a significant decrease in spinal BMD likely indicates true bone loss. In such cases, aggressive management may still be necessary even if total hip BMD has increased. Additionally, rather than implying equivalence or allowing direct comparisons between these two skeletal sites, our study highlights the importance of structured interpretation frameworks to help clinicians make informed decisions when faced with discordant BMD findings.

9. Table 4: BMD and TBS are two different outcomes. Imo, the Table indicates that TBS is less sensitive to changes over time than LS. But that does not mean poor agreement per se. It could indicate, however, that TBS is less suited to evaluate or monitor BMD status.

**Response:** We appreciate the reviewer’s important distinction between BMD and TBS as fundamentally different yet complementary measures—reflecting bone quantity and bone microarchitecture, respectively. In our study, the term “agreement” was used to describe the concordance or discordance in their longitudinal change patterns during follow-up, specifically quantified using Cohen’s kappa statistic. Our intention was not to equate the roles of BMD and TBS, but to highlight the clinical challenge of interpreting discordant changes when both measures are routinely monitored in follow-up assessments.

While TBS is inherently less sensitive to short-term changes, particularly in response to certain treatments, this lower responsiveness may limit its utility for monitoring purposes—especially when clinicians depend on longitudinal trends to guide therapeutic decisions. Our findings underscore the need to better define the role of TBS in monitoring treatment response, rather than suggesting a flaw in the metric itself. We have revised the manuscript on page 12, line 202 and page 1, line 275.

**Page 12, line 202**

Our result was not in reference to direct equivalence between BMD and TBS, but rather to describe the concordance or discordance in their longitudinal change patterns during follow-up.

**Page 16, line 275**

BMD and TBS are fundamentally different but complementary measures—reflecting bone quantity and bone microarchitecture, respectively. According to the literature, increased soft tissue over the lumbar spine can affect both spinal BMD and TBS measurements. While BMD may be influenced, the change typically does not exceed the LSC, and therefore is unlikely to have clinical significance. In contrast, greater soft tissue thickness is associated with a lower TBS value [13, 14]. However, it is believed that TBS is not affected by lumbar degenerative change compared to spine BMD. Dufour et al. reported that osteoarthritis at L4 caused a 19% increase in spine BMD at L4; however, there was no significant change in the TBS [15]. Kolta et al. also demonstrated that lumbar osteoarthritis caused an increase in spinal BMD in patients with lumbar osteoarthritis, whereas TBS was not affected [7]. Additionally, during the follow-up period, there was a significant decrease in the TBS, whereas spinal BMD was unchanged [7]. In our study, there was also a discordance between spinal BMD and TBS in follow-up BMD measurements, with a discordance percentage of 58.0%. From a clinical perspective, discordance between BMD and TBS—such as an improvement in BMD accompanied by a decline in TBS (or vice versa)—can complicate the interpretation of treatment response or changes in fracture risk. Although the ISCD recommends monitoring spinal BMD over TBS [2], the precise role of TBS in tracking the progression of treated or untreated osteoporosis remains uncertain. Generally, anabolic agents produce more pronounced changes in TBS compared to anti-resorptive therapies [5]. Further research is needed to clarify the utility of TBS in monitoring therapeutic response.

10. Conclusion:
“Additionally, the discordance percentage was 43.5% between the monitored spine and the mean bilateral total hip BMD, which indicated poor agreement.”
This statement is not justified. You would never aspect good agreement between both sites.

“We also compared changes between the monitored spinal BMD and TBS in follow-up BMD measurements; the discordance percentage was 58.0%, which also showed poor agreement”
This statement is also not justified. When one measurement is not sensitive to changes over time, while the other is relatively sensitive, I would never expect good agreement.

**Response:** We thank the reviewer for raising this important point and agree that anatomical and physiological differences between measurement sites—and between modalities such as BMD and TBS—naturally limit the expectation of strong agreement. Our intention was not to suggest that perfect or high agreement is expected between the spine and hip, or between BMD and TBS, given their differing sensitivities and biological underpinnings. However, the use of terms such as “poor agreement” in our manuscript was meant to reflect the clinical implications of discordant change patterns. For example, a discordance rate of 43.5% between monitored spine and mean total hip BMD, and 58.0% between spine BMD and TBS, suggests that changes observed at one site or by one method may not reliably reflect changes observed at another—potentially complicating clinical interpretation during follow-up.

We acknowledge the reviewer’s point that such discordance may stem from expected physiological differences and differential responsiveness, particularly in the case of TBS, which is known to be less sensitive to short-term changes. To clarify this distinction, we have revised the manuscript to avoid implying that such discordance necessarily reflects a negative sense, and instead emphasize that these findings underscore the importance of site- and modality-specific interpretation in longitudinal bone health monitoring. We have revised the manuscript on page 13, line 208.

**Page 13, line 208**

In this study, we monitored changes in BMD based on various follow-up BMD measurements. When using the LSC of the mean bilateral total hip BMD, the discordance between the mean bilateral total hip BMD and the total hip BMD of each hip separately was 11.3%. In contrast, when using the average LSC of the right and left total hip BMD, the discordance increased to 19.5%. This suggests that analyzing each hip separately is more sensitive to detecting changes than using the mean bilateral total hip BMD, and the LSC of the mean bilateral total hip BMD is recommended. Additionally, the discordance between the lumbar spine and the mean bilateral total hip BMD was 43.5%, while the discordance between lumbar spine BMD and TBS reached 58.0%, reflecting the differing biological responses and measurement characteristics of these parameters.

11. “When the LSC of the mean bilateral total hip BMD was used to compare the differences in follow-up BMD measurements, the discordant percentage was 11.3%.”
Is this really discordance? When taking a mean of two measurements, it just becomes less sensitive. Thus, a true change in of two hips, may go undetected when taking the mean of two hips. My interpretation is that when high sensitivity is required, it could be more beneficial to monitor and interpret both hips separately.

**Response:** We appreciate the reviewer’s insightful comment regarding the potential insensitivity of using mean bilateral total hip BMD in detecting true changes. We agree that averaging the BMD of both hips may reduce sensitivity, as individual changes in one hip could be attenuated or masked when combined with a stable or minimally changing contralateral hip. This phenomenon may indeed contribute to the observed discordance rate (11.3%). However, our use of the term “discordance” was not intended to imply error, but rather to describe the observed discrepancy in classification outcomes when comparing the monitoring methods between the mean bilateral total hip BMD and the total hip BMD of each hip separately. We agree that this could partly reflect the statistical dampening effect of averaging, and we have revised our discussion to clarify this. Moreover, we concur that in clinical situations where greater sensitivity to localized changes is required—such as in early treatment response or in patients at high risk—it may be more appropriate to monitor each hip separately. This observation further supports the need for tailored monitoring strategies depending on the clinical context. We have revised the manuscript on page 14, line 231.

**Page 14, line 231**

Our results revealed that the discordance percentage was 11.3% between the monitored mean bilateral total hip BMD and the total hip BMD of each hip separately. Among the discordant results, 4.8% of the participants had significantly decreased BMD at one side of the hip and no significant change in the mean bilateral total hip BMD. Additionally, 1.0% of the participants had significantly increased BMD involving one hip and decreased BMD at the other hip, which was not significant when monitoring the mean bilateral total hip BMD. This suggests that analyzing each hip separately is more sensitive to detecting changes than using the mean bilateral total hip BMD. Relying solely on the mean may result in overlooking patients who require more aggressive management. While monitoring the mean bilateral total hip BMD is a simpler and more convenient approach for tracking changes in both hips, it can potentially mask significant changes occurring in one hip. Although this method is appropriate for most patients, in clinical situations where greater sensitivity to localized changes is needed—such as during early treatment response or in high-risk individuals—it may be more appropriate to monitor each hip separately. These findings underscore the importance of adopting tailored monitoring strategies based on the clinical context.

12. The discussion would be strengthened by a more comprehensive integration of the relevant literature.

**Response:** Thank you for your comment. Our aim is to emphasize the need for structured interpretive frameworks that help clinicians understand and respond to discordant findings, rather than to suggest equivalence or direct comparisons between measurement sites or between BMD and TBS. To clarify this distinction, we have revised the manuscript to avoid implying that such discordance is inherently negative. Instead, we highlight that these findings underscore the importance of site- and modality-specific interpretation in the longitudinal monitoring of bone health. This issue is especially relevant in routine follow-ups, where treatment decisions often hinge on the perceived response to therapy. We believe that recognizing this interpretive ambiguity further supports the need for clearer clinical guidance. In response, we have clarified our discussion, incorporated additional relevant literature, and conducted a further round of English language editing to improve clarity and readability. We hope these revisions enhance the scientific contribution of our work.

**Page 12, line 207**

**Discussion**

In this study, we monitored changes in BMD based on various follow-up BMD measurements. When using the LSC of the mean bilateral total hip BMD, the discordance between the mean bilateral total hip BMD and the total hip BMD of each hip separately was 11.3%. In contrast, when using the average LSC of the right and left total hip BMD, the discordance increased to 19.5%. This suggests that analyzing each hip separately is more sensitive to detecting changes than using the mean bilateral total hip BMD, and the LSC of the mean bilateral total hip BMD is recommended. Additionally, the discordance between the lumbar spine and the mean bilateral total hip BMD was 43.5%, while the discordance between lumbar spine BMD and TBS reached 58.0%, reflecting the differing biological responses and measurement characteristics of these parameters.

The 2023 updated ISCD Adult Official Positions recommends that the mean bilateral total hip BMD should be used for monitoring when both hips have been scanned in follow-up BMD measurements [2]. However, they did not mention how to determine the LSC, such as the mean LSC of bilateral total hip BMD or the average LSC of the right and left total hip BMD. In our study, the LSC of the mean bilateral total hip BMD was lower than the average LSC of the right and left total hip BMD. When the LSC of the mean bilateral total hip BMD was used to compare the differences in follow-up BMD measurements, the discordant percentage was 11.3%. In contrast, when the average LSC of the right and left total hip BMD was used, the discordant percentage was 19.5%. Therefore, the mean LSC of the bilateral total hip BMD should be used to monitor the mean bilateral total hip BMD during follow-up BMD measurements.

Our results revealed that the discordance percentage was 11.3% between the monitored mean bilateral total hip BMD and the total hip BMD of each hip separately. Among the discordant results, 4.8% of the participants had significantly decreased BMD at one side of the hip and no significant change in the mean bilateral total hip BMD. Additionally, 1.0% of the participants had significantly increased BMD involving one hip and decreased BMD at the other hip, which was not significant when monitoring the mean bilateral total hip BMD. This suggests that analyzing each hip separately is more sensitive to detecting changes than using the mean bilateral total hip BMD. Relying solely on the mean may result in overlooking patients who require more aggressive management. While monitoring the mean bilateral total hip BMD is a simpler and more convenient approach for tracking changes in both hips, it can potentially mask significant changes occurring in one hip. Although this method is appropriate for most patients, in clinical situations where greater sensitivity to localized changes is needed—such as during early treatment response or in high-risk individuals—it may be more appropriate to monitor each hip separately. These findings underscore the importance of adopting tailored monitoring strategies based on the clinical context.

Chen et al. reported that a diagnosis based on the use of the lumbar spine and one site of hip BMD failed to identify 13.4% to 15.8% of osteoporotic subjects, which may indicate that simultaneously monitoring the spine and both hips represented the best practice for DXA BMD measurements [6]. There was spine-hip discordance between the monitored spine and mean bilateral total hip BMD in follow-up BMD measurements in our study and the discordance percentage was 43.5%. Kolta et al. also reported a significant decrease in the hip BMD, whereas the spinal BMD did not change during a 6-year follow-up [7]. The spine was more affected by age-related degenerative changes than the hip. Liu et al. reported that lumbar osteophytes explained 16.6% of measured changes in the spinal BMD in women [8]. In a study of osteoporotic fractures, women with degenerative lumbar disease showed a 9% to 13% increase in the spinal BMD [9]. Ichchou et al. demonstrated that degenerative lumbar disease could increase spinal BMD by up to 15% in women with severe lumbar osteoarthritis [10]. The causes of the spine-hip discordance may be as follows. First, the peak bone mass and rate of bone loss are not the same for different skeletons [11]. Second, the initial rate of bone loss was greater in the trabecular bone than in the cortical bone in postmenopausal women [12]. Third, spine was more affected by age-related degenerative changes than the hip [8]. Therefore, a significant increase in spinal BMD on repeat testing may result from either true bone gain or degenerative changes. If total hip BMD shows a significant decrease during follow-up, aggressive management may still be warranted despite an apparent increase in spinal BMD. Conversely, a significant decrease in spinal BMD likely indicates true bone loss. In such cases, aggressive management may still be necessary even if total hip BMD has increased. Additionally, rather than implying equivalence or allowing direct comparisons between these two skeletal sites, our study highlights the importance of structured interpretation frameworks to help clinicians make informed decisions when faced with discordant BMD findings.

BMD and TBS are fundamentally different but complementary measures—reflecting bone quantity and bone microarchitecture, respectively. According to the literature, increased soft tissue over the lumbar spine can affect both spinal BMD and TBS measurements. While BMD may be influenced, the change typically does not exceed the LSC, and therefore is unlikely to have clinical significance. In contrast, greater soft tissue thickness is associated with a lower TBS value [13, 14]. However, it is believed that TBS is not affected by lumbar degenerative change compared to spine BMD. Dufour et al. reported that osteoarthritis at L4 caused a 19% increase in spine BMD at L4; however, there was no significant change in the TBS [15]. Kolta et al. also demonstrated that lumbar osteoarthritis caused an increase in spinal BMD in patients with lumbar osteoarthritis, whereas TBS was not affected [7]. Additionally, during the follow-up period, there was a significant decrease in the TBS, whereas spinal BMD was unchanged [7]. In our study, there was also a discordance between spinal BMD and TBS in follow-up BMD measurements, with a discordance percentage of 58.0%. From a clinical perspective, discordance between BMD and TBS—such as an improvement in BMD accompanied by a decline in TBS (or vice versa)—can complicate the interpretation of treatment response or changes in fracture risk. Although the ISCD recommends monitoring spinal BMD over TBS [2], the precise role of TBS in tracking the progression of treated or untreated osteoporosis remains uncertain. Generally, anabolic agents produce more pronounced changes in TBS compared to anti-resorptive therapies [5]. Further research is needed to clarify the utility of TBS in monitoring therapeutic response.

The strength of this study lies in the inclusion of a substantial number of participants who underwent follow-up BMD measurements of the spine and both hips. Second, to the best of our knowledge, this is the first study to compare different methods for monitoring BMD changes. Nevertheless, this study had some limitations. First, it was conducted in postmenopausal Asian women, potentially affecting the generalizability of our results. Second, BMD and TBS were measured using Hologic instruments only. However, further studies are required to confirm these findings.

In conclusion, our study demonstrated that analyzing each hip separately is more sensitive for detecting changes than using the mean bilateral total hip BMD. When monitoring the mean bilateral total hip BMD on repeat tests, it is recommended to use the LSC of the mean bilateral total hip BMD. Discordance between spine and hip BMD, as well as between spinal BMD and TBS, was observed in follow-up measurements. These findings emphasize the need for structured interpretation frameworks to guide clinical decision-making in the presence of discordant results.
